# Detection of Genomic Variation by Selection of a 9 Mb DNA Region and High Throughput Sequencing

**DOI:** 10.1371/journal.pone.0006659

**Published:** 2009-08-17

**Authors:** Sergey I. Nikolaev, Christian Iseli, Andrew J. Sharp, Daniel Robyr, Jacques Rougemont, Corinne Gehrig, Laurent Farinelli, Stylianos E. Antonarakis

**Affiliations:** 1 Department of Genetic Medicine and Development, University of Geneva Medical School, Geneva, Switzerland; 2 Ludwig Institute for Cancer Research and Swiss Institute of Bioinformatics, Bâtiment Génopode, Lausanne, Switzerland; 3 Bioinformatics and Biostatistics Core Facility, School of Life Sciences, Ecole Polytechnique Fédérale de Lausanne (EPFL), Lausanne, Switzerland; 4 Swiss Institute of Bioinformatics, Lausanne, Switzerland; 5 FASTERIS SA, Geneva, Switzerland; University of Montreal, Canada

## Abstract

Detection of the rare polymorphisms and causative mutations of genetic diseases in a targeted genomic area has become a major goal in order to understand genomic and phenotypic variability. We have interrogated repeat-masked regions of 8.9 Mb on human chromosomes 21 (7.8 Mb) and 7 (1.1 Mb) from an individual from the International HapMap Project (NA12872). We have optimized a method of genomic selection for high throughput sequencing. Microarray-based selection and sequencing resulted in 260-fold enrichment, with 41% of reads mapping to the target region. 83% of SNPs in the targeted region had at least 4-fold sequence coverage and 54% at least 15-fold. When assaying HapMap SNPs in NA12872, our sequence genotypes are 91.3% concordant in regions with coverage≥4-fold, and 97.9% concordant in regions with coverage≥15-fold. About 81% of the SNPs recovered with both thresholds are listed in dbSNP. We observed that regions with low sequence coverage occur in close proximity to low-complexity DNA. Validation experiments using Sanger sequencing were performed for 46 SNPs with 15-20 fold coverage, with a confirmation rate of 96%, suggesting that DNA selection provides an accurate and cost-effective method for identifying rare genomic variants.

## Introduction

The completion of a human genome reference sequence has opened new horizons for genetic analyses and biomedical research [1], [2]. Recent advances in sequencing technologies now permit the resequencing of individual genomes. A total of five individual human genomes have been published to date. J.C. Venter's genome was sequenced using the Sanger method [3], while J. Watson's genome was completed using 454 massively parallel sequencing and cost one hundredth [4] of the Venter's genome [5]. The three genomes of Yoruban and Asian individuals, as well as a leukaemia genome were sequenced by a massively parallel approach using reversible terminator chemistry (Illumina-sequencing [6], [7], [8], [9]). Despite these advances, the cost of sequencing a human genome remains high for routine diagnostic purposes.

Applications such as mapping and identifying disease-associated regions do not require the sequencing of an entire genome. The ability to genotype common polymorphisms with SNP arrays has greatly enhanced the mapping of disease-associated loci [10]. Recent studies have demonstrated the potential power of resequencing candidate genes to detect rare variants underlying disease-associated traits or to identify somatic mutations associated with tumors [11], [12], [13], [14], [15], [16], [17]. However, *de novo* discovery of pathogenic mutations for inherited or sporadic disease is limited by the low throughput of current targeted sequencing methodologies.

For many genetic diseases the genomic interval of a causative mutation is localized using linkage and association studies [18], [19], [20]. To allow cost-effective resequencing of these loci, recent studies proposed a microarray-based method for selection of a portion of a genome that could then be sequenced. This method exploits tiling oligonucleotide arrays to select specific regions of genomic DNA by hybridization, and the enriched sample is subsequently sequenced by high throughput methods. This approach was successfully applied to the sequencing of both discontinuous exonic sequences, and contiguous genomic regions [21], [22], [23]. Alternatively, DNA capture trials have also been performed using in-solution hybridization to biotinylated BAC or RNA probes [24], [25].

The goal of this study was to demonstrate the feasibility to sequence a selected fraction of a genome at low cost employing custom design tiling arrays and high throughput sequencing. The repeat-masked region of 8.9 Mb corresponding to a contiguous sequence of 15 Mb was targeted on human chromosomes 21 and 7. We demonstrate that this method can be applied to accurately define sequence variants in contiguous stretches of genomic DNA and could be used to identify pathogenic mutations in patients.

## Methods

### Sample preparation

Genomic DNA isolated from a lymphoblastoid cell line of HapMap individual NA12872 was used in this study. 20 ug of DNA in 500 ul H_2_O was sonicated on ice using a Branson 450 sonifier (10% amplitude, 150 seconds, using pulses of 0.5 sec on, 0.5 sec off). The length of sonicated fragments varied between 100 and 700 bp. The library size was estimated by electrophoresis in a 2% agarose gel. DNA was precipitated with salt/isopropanol.

End filling of DNA breaks was performed in 200 ul (18U T4 DNA Polymerase, 60U T4 Polynucleotide Kinase, NEB T4 DNA Polymerase Buffer with BSA, 1 mM dNTP, 0.5 mM ATP (New England Biolabs)) for 20 minutes at 12°C, 20 minutes at 25°C, and 20 minutes at 75°C. DNA was purified with phenol∶chloroform∶isoamyl alcohol (25∶24∶1) and precipitated with salt/isopropanol. A-tailing was performed in a volume of 50 ul (5U Taq polymerase, 1x PCR buffer, 4 uM dATP, (New England Biolabs)) for 45 min at 72°C. Y-tailed Illumina adapters were used, that allow amplification from 2 different primers complementary to single strand tails of the adapter (Genomic DNA/ChIP-Seq oligonucleotide sequences at http://intron.ccam.uchc.edu/groups/tgcore/wiki/013c0/Solexa_Library_Primer_Sequences.html). Sticky end ligation was performed on the A-tailed sample DNA using T4 DNA ligase (New England Biolabs) and a 6-fold molar excess of Y-tailed adapters with 3′ T overhang according to the manufacturer's protocol. The sample was PCR amplified in four 50 ul reactions using standard Illumina primers and the following conditions: 95°C for 2 minutes, followed by 30 cycles of 95°C for 30 seconds, 63°C for 30 seconds and 72°C for 60 seconds, then at 72°C for 5 minutes.

20 ug of amplification products were precipitated and mixed with 100 ug COT-1 DNA (Roche). Amplified samples were hybridized to capture arrays for approximately 60 h at 42°C with active mixing using a MAUI hybridization station. After hybridization, arrays were washed at room temperature for 2 minutes with Stringent Wash Buffer, 2 mins with Wash Buffer I, 1 min with Wash Buffer II, and 15 seconds with Wash Buffer III (Nimblegen). Slides were dried, and captured DNA fragments were eluted on a hotplate at 95°C using two 250 ul aliquots of water. Eluted DNA was lyophilized and amplified using the same primers, and the same conditions as above.

A custom array design produced by Nimblegen (www.nimblegen.com) was used. Probe design was performed using the repeat-masked sequence of human chromosome 21 (hg17) and ENCODE region ENm001, comprising a total length of 8.9 Mb (7.8 Mb of chr21 and 1.1 Mb of ENm001). In total, 385,000 probes with an average length of 50 bp were used to cover the entire region, with an average density of one probe per 22 bp. The array design is accessible from MIAMExpress database (http://www.ebi.ac.uk/miamexpress/) with the accession number A-MEXP-1601.

High throughput single-end sequencing was performed using the Illumina GA-II instrument. 36 cycles of reversible terminator chemistry reaction were performed with sequencing kit- SBS v 2.0, tiles – 100. For the initial handling of the data standard Illumina Analysis Pipeline GAPipeline-1.0rc4 was used.

Parallel experiments on the same array design were conducted, with and without addition of COT-1 DNA. For the enrichment without COT-1 one lane of Illumina flow-cell was run, and for the experiment with COT-1, two such lanes were run.

The whole experiment including library preparation, hybridization, sequencing and analysis of polymorphisms could be performed within two weeks. The total cost of the experiment is mainly comprised of the cost of the array and sequencing.

### Sequence analysis

We first took the set of reads produced by the Illumina pipeline and determined using fetchGWI software [26] the reads with an exact match on the UCSC hg18 reference genome. The results, presented in Table 1, indicate that only 13% of the reads find an exact match, and 85% of the reads exhibit variants or sequencing errors. We then used Rolexa software [27] to improve the quality of the reads. The Rolexa software pipeline utilizes the image intensity files produced by the Illumina sequencing equipment and performs base calling to produce the sequence reads. The software accept parameters that determine how stringently the nucleotides are discriminated between fully qualified bases (A, C, G, and T), or degenerate bases (using the IUPAC codes) when the intensity files indicate that the probabilities associated with each of the 4 possible nucleotides do not allow an unambiguous base calling. We performed 3 runs of the Rolexa software, each with a different set of parameters. Results are shown in table 1. We used the reads produced by the relaxed parameter set since the number of reads with no exact matches fitted the best to our expectation of ∼45% that was calculated based on the Illumina error rates and expected frequency of SNPs. As the basic error rate in Illumina reads is ∼1%, approximately one third of the 36 nt reads will contain an error, so about 33% of the reads are expected to not match exactly the reference sequence. Including SNPs in this calculation, some predictions estimate ∼1 SNP per 300 nt. Assuming that reads are placed randomly in the genome, we expect the reads to start at each possible nucleotide along the sequence. Since the reads are 36 nt in length, for each SNP we will have 36 different reads that contain the SNP and thus will not match exactly to the reference genome, while the remaining 264 starting positions along a 300 nt stretch will match exactly, equating to ∼12% of reads containing mismatches due to SNPs. In total, we thus expect 33%+12% = 45% of reads with mismatches.

**Table 1:** Number of sequence reads extracted from intensity files using Illumina and Rolexa analysis.

Method	Total reads	Uniquely mappable reads/bases	% of reads	Reads/bases with multiple genomic matches	% of reads	Missed reads/bases	% of reads
Illumina default pipeline	33,280,140	4,335,125/155,820,008	13	779,445/27,968,664	2	28,165,570/1,002,508,008	85
Rolexa default parameters log2(c(1.5,2.5,3.5))	10,217,510	5,430,218/165,755,132	53	1,189,132/33,511,489	12	3,598,160/100,396,369	35
Rolexa strict parameters (log2(c(1.05,2.5,3.5)))	4,138,591	2,789,546/78,062,584	67	555,370/14,964,244	14	793,675/21,821,150	19
Rolexa relaxed parameters (log2(c(1.9,2.9,3.9)))	23,022,818	4,109,921/132,362,857	18	830,843/25,786,098	4	18,082,054/545,943,814	78

The Rolexa produces reads of different length, between 25 nt and 36 nt.

A total of 23 million reads with sufficient length and quality were obtained. 4,109,921 reads found a unique match (group U of unique reads), 830,843 reads found multiple matches (group R of repeated reads), and 18,082,054 reads remained with no match (group M of missed reads). 1,751,305 of the U reads (42%) were mapped to the target region (1,605,903 unique reads originate from HSA21 and 143,407 reads – from HSA7). 2,358,616 unique reads originate from non-target regions. The file containing Rolexa raw reads is accessible at the http://home.adm.unige.ch/~nikolaev/Rolexa_relaxed.rar. Enrichment was calculated as a ratio of the number of reads with a match within the selected region divided by the length of the region, and the number with a match outside the selected region divided by the length of the rest of repeat-masked portion of the genome.

The regions with the low sequence coverage and the regions from the third quartile of the coverage distribution were selected in a way that at least 80% of their length should be comprised of the nucleotides covered less than 3 fold and between 16 and 29 fold respectively. Low-complexity DNA was detected using the dust program [28] with a parameter -10.

In order to discover polymorphisms in the sequenced regions, reads with no exact match on the reference genome (group M) were analyzed to determine their best alignment to the genome. We have found a number of limitations of Maq [29] and Eland [7] aligners such as the incapability of processing reads of different lengths simultaneously, that precludes using these programs for processing the Rolexa output. Rolexa reads were therefore aligned to the reference genomic sequence using the global alignment software align0 using the method described hereafter.

First we selected from each group of reads (U, R, and M) those that have a high chance of finding a good quality alignment with the chromosomal regions of interest by requiring that they share at least a common 12-mer with the region, using the tagger tool from the fetchGWI software package.

SNPs were predicted when an observed difference between the reads and reference sequence occurred at a sufficient rate among all the reads that cover the position. In this case we required at least 4 reads to call a SNP and that the minor allele is present in at least 20% of reads.

## Results and Discussion

### Enrichment for the targeted DNA sequences

**Figure 1:**
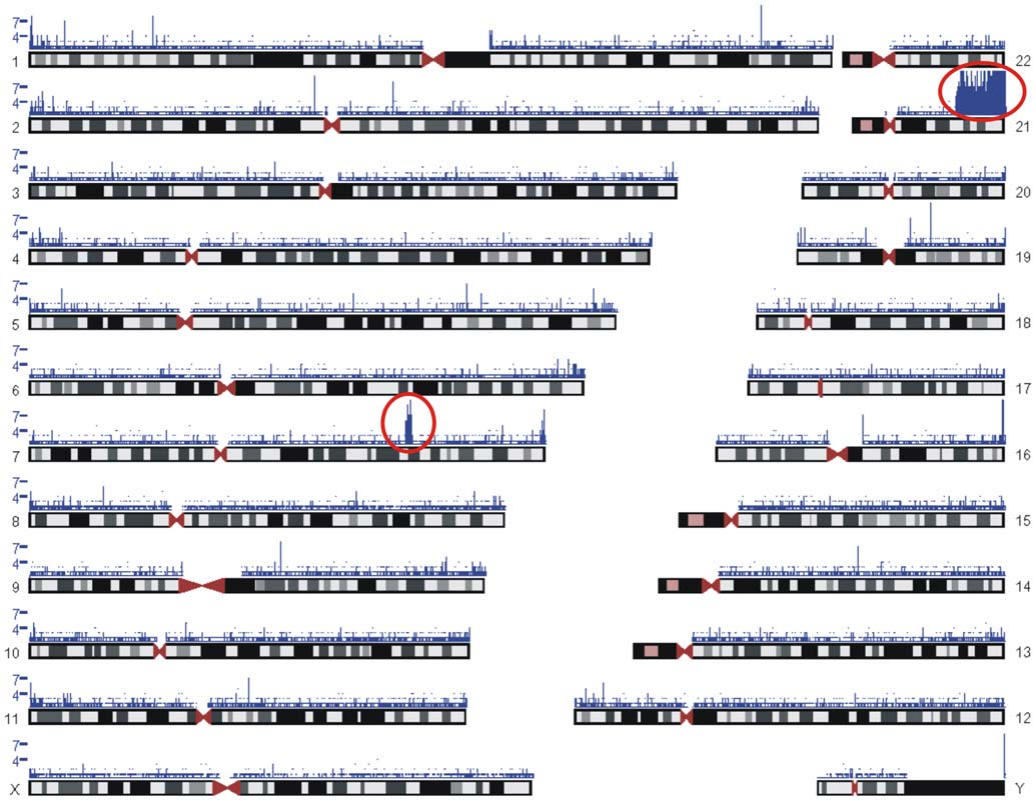
Enrichment of target regions. All sequencing tags that map uniquely to the reference human genome are displayed in blue. The height of peaks corresponds to the number of tags mapping to the same location. Red circles represent genomic areas plotted on the array. The image was generated using Genome Graphs (http://genome.ucsc.edu/cgi-bin/hgGenome).

In order to characterize sequence variants in a genomic region of interest, we performed microarray-based enrichment using genomic DNA from HapMap individual NA12872. The selected region includes 8.9 Mb of non-repetitive DNA from human chromosome 21 (31.6 Mb–46.9 Mb) and chromosome 7 (115.3 Mb–117.2 Mb). A sample from HapMap individual was chosen in order to compare the variants identified in this study by sequencing with those genotyped previously [30]. The enriched sample was sequenced using an Illumina GAII instrument, and signals extracted using both the Illumina default output and our own Rolexa analysis [27]. The selection procedure resulted in 260-fold enrichment of the target region when the sample was mixed with COT-1 DNA at the hybridization step. Figure 1 displays all 4.1 million sequence tags recovered by Rolexa that map unambiguously to the reference genome. The high density of reads on HSA21 corresponds to the target region plotted on the capture array, and the second enrichment peak on HSA7 corresponds to ENCODE region ENm001.

We checked if unique reads from outside the target region cluster in the genome. The crude analysis have shown that the average coverage is similar in all chromosomes (mean = 0.03 and standard deviation = 0.007) suggesting that such reads do not originate from a specific locus in a genome. We found out that only 5% of unique reads from outside the target region comprise clusters. 3594 clusters with an average coverage of more than 1 and the region length over 200 bp were detected. 78% of these regions overlap to repeats, and 3% overlap to the regions of segmental duplications longer than 1000 bp. Out of 184 segmental duplications from the target region of HSA21 we observe increased sequence coverage for 95 outside the target region. The challenging problem at the enrichment step is to deplete the repeated elements. The presence of repeats in the enriched sample diminishes the specificity of sequencing of the target region and increases the sequencing cost. After the COT-1 hybridization the number of reads that correspond to repeats vary from 15% to 40% of all mappable reads depending on the signal extraction criteria and blast parameters.

### Optimization of the microarray-based genomic enrichment

We optimized the enrichment protocol in order to improve the specificity of sequencing. Sonicated DNA was ligated with Y-tailed adapters that include sequencing primers. The sample material was amplified using Illumina primers to generate sufficient quantities required for hybridization. When the amount of starting material is limited, Whole Genome Amplification before sonication could provide the necessary DNA quantity.

The sample DNA was mixed with a five-fold excess of COT-1 DNA in order to reduce the proportion of non-specific hybridization of repetitive DNA. After elution of the hybridized fraction of the sample we performed PCR with primers complementary to the adapters in order to restore double stranded library and to reduce the relative abundance of COT-1 DNA.

**Figure 2:**
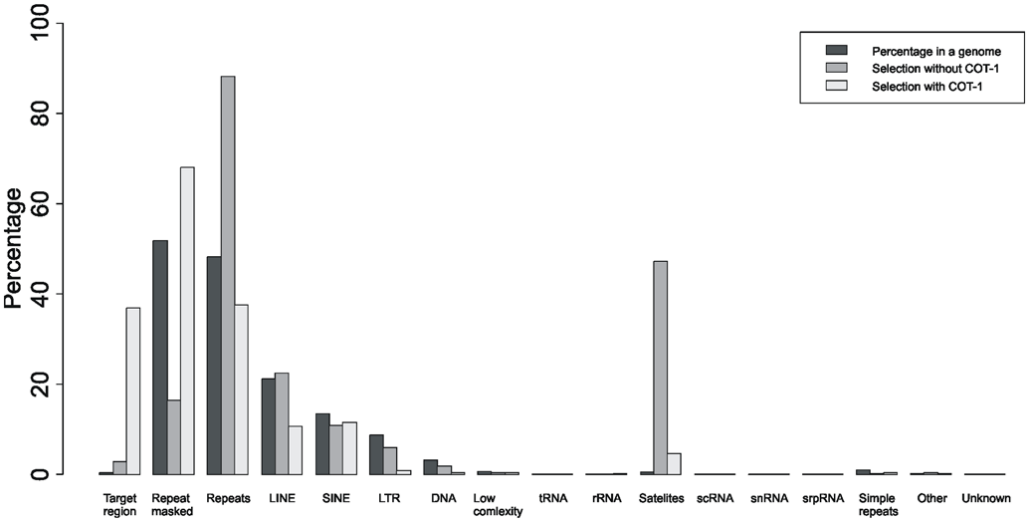
Proportion of different genomic elements in the captured samples with and without COT-I DNA as compared to their relative abundance in the human genome. Reads were mapped to the reference human genome using BLAT, allowing 2 mismatches and 1 indel with a minimal perfect match of 30 bp.

Next we compared the results of hybridizations with and without COT-1 DNA. In order to better understand the impact of COT-1 DNA on the enrichment we assessed how efficiently it competes for hybridization with repetitive elements. We compared the proportion of Illumina reads that map to different kinds repeats (with and without COT) to the proportion of these elements in a genome (Fig. 2). Generally, addition of COT-1 DNA during array hybridization reduced the proportion of repetitive elements present in the captured DNA by 33%, thereby increasing the percentage of reads from non-repetitive genomic elements, and improving enrichment for the target sequence ∼14-fold.

More specifically, addition of COT-1 reduced the proportion of LINE elements in the enriched sample by 2-fold and the proportion of Satellite repeats by 10-fold. COT-1 did not significantly reduce the amount of SINEs. However, contrary to the other repetitive elements, after array enrichment and sequencing the proportion of satellite sequences was still substantially higher than that expected compared to their prevalence in the genome. With the current enrichment, out of all reads that could be mapped back to the human genome, satellites comprise 4.5%, LINEs 10.5%, and SINEs 11.4% (in the genome satellites comprise 0.45%, LINEs - 21.1% and SINEs – 13.5%). Apart from repeats we also observed that 26% of uniquely mappable reads obtained after enrichment were located outside the target region. A possible explanation for their abundance is that random species of sample DNA non-specifically anneal to the single stranded DNA hybridized to the array probe by the primer, which in our case is 57 bp. This length of the primer might be sufficient to form a stable double stranded DNA hybrid; shorter primers however, may further improve the selection procedure. Further improvements to the enrichment could be made with excess of primers to the hybridization mix to saturate the single stranded ends of the sample DNA.

### Standard Illumina pipeline vs. Rolexa

The Rolexa algorithm uses a probabilistic base-calling method which allows detection of low-quality bases, assigning them either as ambiguous sites, or alternatively trimming the read length. We compared the final sequence assembly generated by extraction of sequence signals using the standard Illumina output (GAPipeline-1.0rc4 for LF) against that generated using the Rolexa algorithm [27]. Using Rolexa default paremeters, the number of reads with a unique perfect match in the reference genome increased by 20% compared to the standard Illumina pipeline (5,430,218 versus 4,335,125, Table 1). The enrichment efficiency for our target sequence remained almost the same in the two algorithms (255-fold enrichment for Illumina versus 260-fold for Rolexa).

**Figure 3:**
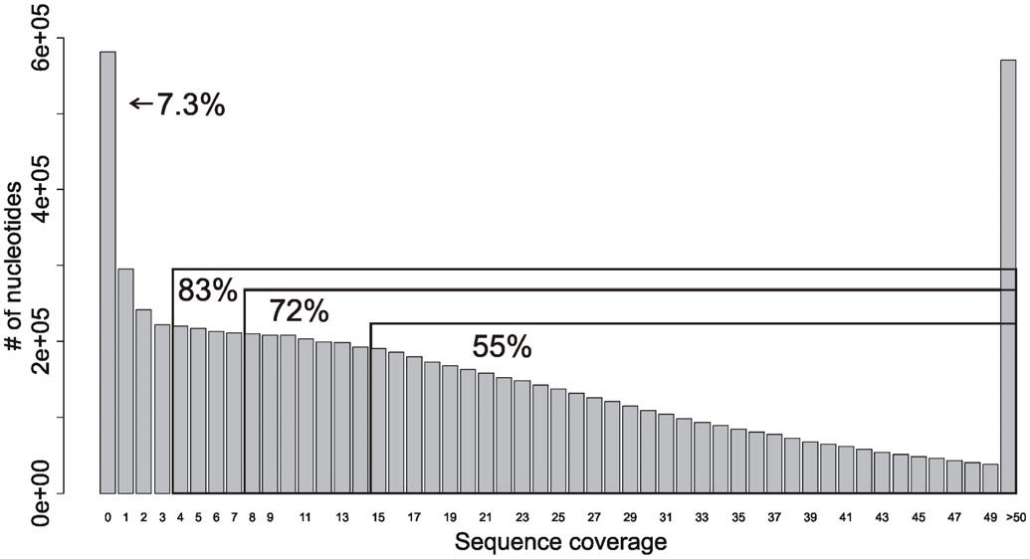
Distribution of nucleotide coverage in the targeted 7.8 Mb region of chromosome 21. The numbers in the boxes are the percentages of the target region covered more than 4, 8 and 15 folds.

We observed that out of all mappable reads to the reference genome, about 42% correspond to the target region, equating to a 16-fold median coverage, interquartile range 6- to 29-fold. A total of 7.3% of the target region had no coverage, while 83% had at least 4-fold coverage, and more than a half of the target region is covered at least 15 times (Fig. 3). Previous estimates [7] suggest that a read depth of 15-fold could identify ∼97% of SNPs.

**Figure 4:**
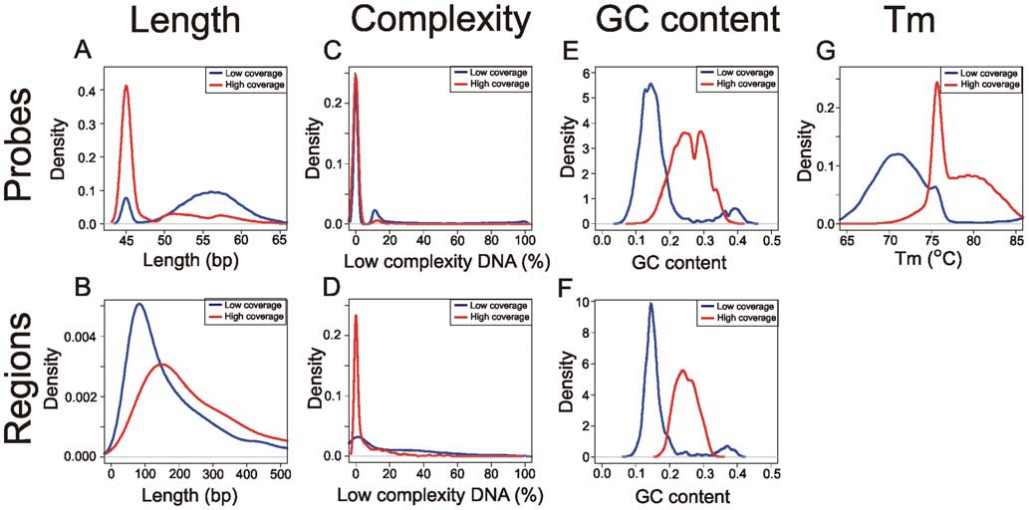
Distribution of sequence features of regions and corresponding probes with low (<3 fold) and normal (15-29 fold) sequence coverage. A,B) Length of the regions/probes. C,D) Percentage of low complexity DNA inside the regions/probes. E,F) GC content of regions/probes. G) Hybridization temperature of the probes.

In order to identify factors influencing the variation in sequence coverage between different genomic regions, we analyzed the sequence and probe properties of the target region. We divided our target loci into two groups: regions with less than 3-fold coverage, and with 16–29 fold coverage (corresponding to the third quartile of the sequence coverage distribution) (Fig. 4). We observed that regions with low coverage are shorter (median = 139 bp) than regions with the good coverage (median = 227 bp) (Fig. 4b). The Nimblegen probes contain almost no low complexity DNA on the array (Fig. 4c), but we observed that the regions of low coverage contain much more low complexity DNA compared to the regions with the good coverage (Fig. 4d). Next we observed that regions of low coverage and corresponding probes are AT rich relative to the genome average (median GC content = 0.3, genome average = 0.41) (Fig. 4e,f). As the array design used in this study is isothermal, and adenine-thymine (A-T) base pair have lower energy than guanine–cytosine (G-C) base pair, AT rich probes are longer (median length = 56 bp) than in regions which yield good sequence coverage (median = 45 bp, Fig. 4a). This also results in a lower mean annealing temperature of probes in the low coverage regions (median = 71.6°C) compared to the regions with the good coverage (median = 77.5°C, Fig. 4g).

We also observed that regions of low-coverage are associated with low-complexity DNA. We hypothesized that this phenomenon may be an artifact resulting from the inability to uniquely map short Illumina reads from these regions to the Human Genome. To test this, we re-mapped reads that match multiple genomic loci (group R) to the regions of low-coverage, this time randomly assigning ambiguously mapping reads to one of their genomic matches. We observed that the coverage increased to a median of 4-fold (interquartile range 2–6 fold), with a maximal coverage of 69-fold. After this step, only four regions in our target loci remained without coverage (criteria of less than 3 fold coverage over 80% of the region). Thus we conclude that poor coverage for some genomic loci does not result from an inability to capture these loci with the array design used, but instead is attributable to the difficulty of uniquely mapping short sequence reads back to the reference genome in regions of reduced complexity. Protocols for paired-end sequencing at 75 bp may reduce the proportion of reads that have multiple matches in the genome and originate in low-complexity DNA regions. This problem can also be reduced with the use of mapping algorithms that do not discard reads without a unique genomic match.

### SNP detection

**Figure 5:**
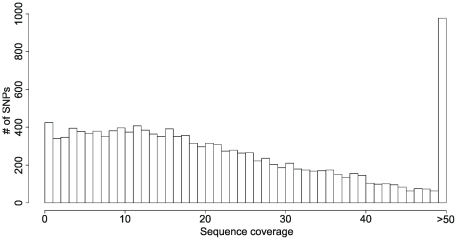
Histogram of dbSNP positions coverage on chromosome 21.

**Figure 6:**
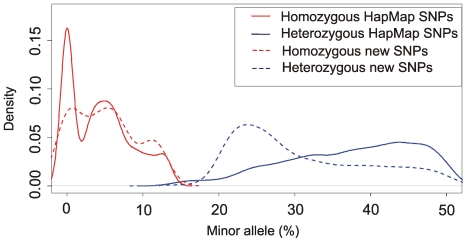
Distribution of percentages of a minor variant for SNPs as recovered by Illumina sequencing. Red: homozygous SNPs, blue: heterozygous SNPs. Solid line: HapMap SNPs, dashed line: new SNPs.

We next performed SNP and indel calling using fetchGWI [26], align0 [29], and perl scripts. In order to extract SNP information from the target region we selected a threshold for SNP calling such that a variant should be seen in at least in 4 independent reads. Thus we required a minimal coverage of 4-fold for homozygous SNPs and 8-fold for heterozygous SNPs. Using these criteria we were able to extract data for homozygous SNPs from 83% of the target region (6.5 Mb) and heterozygous SNPs from 72% of the region (5.6 Mb), recovering a total of 9025 putative variants (3212 homozygous and 5797 heterozygous, Table 2, Fig. 5). A total of 81% of the identified variants are present in dbSNP (www.ncbi.nlm.nih.gov/projects/SNP/). The same type of analysis conducted in human individual genomes have shown that in the Watson genome 82% of all variants were present in dbSNP, 74% in a Yoruban genome, and 86% in an Asian genome [5], [7], [8]. Thus our analysis is consistent with expectations from previously sequenced genomes, suggesting the false positive rate after sequence capture is similar to previous whole-genome studies. We were able to confirm 91.3% of all SNPs identified in NA12872 by the HapMap study, suggesting a low false negative rate in our analysis (Table 3). In order to identify new SNPs in the target region, we first examined the distributions of heterozygous and homozygous variants from dbSNP (Fig. 6). We then set a threshold for assigning a SNP to a hetero- or homozygous state based on the intersection of the distributions, corresponding to 14% of minor allele frequency (below this threshold the SNP was considered homozygous, and above heterozygous). Interestingly, while 36% of SNPs already present in dbSNP were identified as homozygous in NA12872, out of the 1659 new SNPs we identified, only 8% were homozygous. This observation can best be explained if many of the novel SNPs identified are rare variants that are therefore unlikely to be found in the homozygous state. This hypothesis predicts that the shape of the distribution of allele percentages in new SNPs should be the same as in the training set. Indeed this is the case for homozygous SNPs, but for heterozygous SNPs it is different (Fig. 6). This suggests that a fraction of putative new heterozygous SNPs we detect might be false positives.

**Table 2:** Numbers of homozygous and heterozygous SNPs identified in NA12782 at different sequence coverage thresholds.

Categories of SNPs	All SNPs	Heterozygous SNPs	Homozygous SNPs
All SNPs identified with ≥4-fold coverage	9009	5797	3212
All SNPs identified with ≥15-fold coverage	6090	4224	1866
dbSNPs identified with ≥4-fold coverage	7350	4302	3048
dbSNPs identified with ≥15-fold	4846	3081	1765
HapMap SNPs identified with ≥4-fold coverage	3788	2122	1666
HapMap SNPs identified with ≥15-fold coverage	2810	1745	1065
New SNPs identified with ≥4-fold coverage	1659	1495	164
New SNPs identified with ≥15-fold coverage	1244	1143	101

**Table 3:** Comparison of SNPs recovered in this study using different thresholds of sequence coverage (4- and 15-fold) with HapMap SNPs.

Categories of SNPs	4 fold	%	15 fold	%
All tested dbSNPs from the target region	13147	100	7930	100
Concordant SNPs	12009	91.3	7765	97.9
Discordant SNPs (homozygous in HapMap)	139	1.1	63	0.8
Discordant SNPs (heterozygous in HapMap)	999	7.6	102	1.3

In the part of the target region that is covered at least 15 times we detect 137 indels, among those, 65 indels overlap to dbSNP positions and 17 are annotated in NA12872. Thus 53% of detected indels are novel. The median indel length obtained is 8 bp. Interestingly, 39% of detected indels are heterozygous. The frequency of indels observed is 1.8–3 times smaller than recovered in the two individual genomes sequenced with Illumina technology [7], [8].

Lowering the coverage threshold to 3 increases the overlap between our data and dbSNP from 91.3% to 93.7%. However, the number of putative variants increased from 9025 to 27616, suggesting that this relaxed threshold results in a dramatic increase in the false positive rate.

Previous *de novo* sequencing studies have suggested a minimum of 15-fold sequence coverage is required for robust identification of heterozygous SNPs [7]. Based on this observation, we selected 6090 homozygous and heterozygous SNPs from the target region with at least 15-fold coverage. 60% of all dbSNP positions located in the target region represented on our enrichment array were present at or above this threshold. With this coverage threshold we correctly identified 97.9% of dbSNPs, 6.6% better compared to when a minimum coverage of 4 fold was used.

The target region contains 153 annotated genes on chromosome 21 (excluding the family of *KRTAP10* genes). We observed 162 SNPs in the Coding Sequences (CDS), among those 58 were non-synonymous (Table 4). On average we detect 1.1 SNPs per gene CDS a value concordant with previous estimations where this value varies from 0.5 to 3.7 [31], [32]. The proportion of new variants among coding SNPs is roughly the same as among all detected SNPs ∼20%. 37% of all coding SNPs are homozygous; this is higher than that observed for all SNPs (∼30%).

**Table 4:** SNPs in the coding sequences of the genes from the target region.

Categories	All SNPs	Synonymous SNPs	Non-Synonymous SNPs
All coding SNPs	162	85	77
dbSNPs	130	72	58
new SNPs	32	13	19
homozygous	60	31	29
heterozygous	103	54	49

### SNP validation

106 SNPs and 30 indels detected in this study were selected for validation by Sanger sequencing. We were able to confirm 80% of all SNPs and 87% of indels (Table 5). In order to assess how the depth of coverage impacts on the accuracy of SNP detection, we divided the selected SNPs into two categories: (1) those with 15-40-fold coverage, and (2) those with>40-fold coverage. 96% of SNPs from the first group are confirmed, while the rate of false positives in the second group is much higher, reaching 32%. The high rate of false positives among highly covered SNPs might be explained by non-specific hybridization of low-complexity DNA. Thus the variants identified in low-complexity DNA should be treated with caution since they contain many false positives.

**Table 5:** SNP validation with Sanger sequencing.

	All SNPs				New SNPs				dbSNPs				Indels			
	Confirmed	FALSE	%False	all	Confirmed	FALSE	%False	all	Confirmed	FALSE	%False	all	Confirmed	FALSE	%False	all
All SNPs	85	21	19.8	106	37	16	30.2	53	48	5	9.4	53	26	4	13.3	30
15–40 fold	44	2	4.3	46	18	2	10.0	20	26	0	0.0	26	-	-	-	-
>40 fold	41	19	31.7	60	19	14	42.4	33	22	5	18.5	27	-	-	-	-
35–50% het	-	-	-	-	24	5	17.2	29	-	-	-	-	-	-	-	-
20–30% het	-	-	-	-	10	9	47.4	19	-	-	-	-	-	-	-	-
0–14% hom	-	-	-	-	5	0	0.0	5	-	-	-	-	-	-	-	-

“False” – number of false positives, “% False” – percentage of false positives. “15–40 fold” and “>40 fold” - indicate the groups of SNPs with different sequence coverage. “35–50% het”, “35–50% het” and “0–14% hom” – indicate the groups of heterozygous and homozygous putative new SNPs with different percentages of the minor allele. “-” – the value is not calculated.

Among tested SNPs, 90% of new SNPs and 100% of dbSNPs with15-40-fold coverage are confirmed by Sanger sequencing. This suggests a high SNP prediction potential of the method.

Ideally for heterozygous SNPs, each allele should be present in 50% of overlapping reads, and for homozygous SNPs all reads should show the same nucleotide. Practically, the distribution of the alleles in our experiment is different, as shown in Figure 6. We selected heterozygous SNPs which were present in (i) 35–50% of reads, and (ii) 20–30% of reads, and homozygous SNPs which were observed in 86–100% of overlapping reads. As expected, heterozygous SNPs with allelic ratios closer to 50∶50 have a false positive rate 2.8 times lower than SNPs with allelic ratios in the range 20∶80 to 30∶70. All homozygous SNPs tested were confirmed.

We conclude that microarray based selection followed by Illumina sequencing is a powerful technique for *de novo* SNP and short indel identification in specific genomic loci. Our analysis efficiently recovers SNPs from the genomic loci with the normal sequence complexity. Interestingly, the presence of low complexity DNA inside the target region results in reduced sequence coverage because of the difficulty to map uniquely the short Illumina reads and will require additional efforts in order to produce sufficient sequence coverage required for accurate SNP identification. Conversely, regions of unusually high sequence coverage also often correspond to low complexity DNA that exhibits an increased rate of false positive SNPs, and which should therefore be treated with caution. Further we show that putative heterozygous SNP positions that are present at the expected 50∶50 allelic ratio are the most robust predictions with lowest false positive rate.

Given that the current cost of whole genome sequencing remains high for routine analysis of large populations, techniques that allow targeted sequencing of defined genomic regions are valuable tools to facilitate the search for causative mutations in genetic disease cases when prior information regarding the location of a mutation is available. Continuing increases in throughput will further decrease the cost of targeted resequencing.
